# Additive Manufacturing of Engineered Tissue Constructs: Current Strategies and Future Directions

**DOI:** 10.3390/bioengineering13050562

**Published:** 2026-05-16

**Authors:** Alexander Yu. Prosekov, Daria V. Titarenko, Marina G. Kurbanova, Oksana V. Smolovskaya, Oksana V. Kozlova

**Affiliations:** 1 Laboratory of Biocatalysis, Kemerovo State University, Kemerovo 650000, Russia; rector@kemsu.ru; 2 Technological Institute of Food Industry, Kemerovo State University, Kemerovo 650000, Russia; kurbanova-mg@mail.ru (M.G.K.); ms.okvk@mail.ru (O.V.K.); 3 Institute of Biology, Ecology and Natural Resources, Kemerovo State University, Kemerovo 650000, Russia; smol_vet@mail.ru

**Keywords:** 3D bioprinting, tissue engineering, regenerative medicine, bioinks, additive manufacturing

## Abstract

The advancement of modern regenerative medicine is closely associated with additive technologies that enable the creation of tissue-engineered constructs and personalized bioprostheses. Three-dimensional bioprinting allows precise modeling of tissue architecture and extracellular matrix microstructures. Recent studies demonstrate rapid growth in the use of 3D bioprinting for biomedical applications including regenerative medicine, pharmaceutical research, and biotechnology. Special attention is given to the development of bioinks that combine biological and structural functions and maintain cell viability during printing. Modern technologies allow the fabrication of skin, bone, vascular, and cartilage tissues with high structural accuracy. The technology is also actively used in reconstructive surgery for the production of personalized implants. However, challenges remain related to vascularization, standardization of materials, and ethical aspects of clinical use. This review summarizes the main principles of 3D bioprinting, technological approaches, biomedical applications, and future perspectives of additive technologies in regenerative medicine.

## 1. Introduction

Three-dimensional printing is an additive manufacturing technology that enables the creation of spatial structures through the controlled layer-by-layer deposition of material. In the biomedical field, this approach is increasingly used for the fabrication of functional tissue constructs and organ substitutes based on living cells and biomaterials. The development of such technologies has significantly expanded the possibilities of tissue engineering, allowing the production of structures that replicate the architecture and biological characteristics of native tissues. Many researchers consider the fabrication of patient-specific prosthetic and biological constructs using 3D printing technologies to be one of the most promising directions in modern regenerative medicine [1].

The bioprinting workflow typically includes several consecutive stages: digital modeling, preparation of biological materials, controlled fabrication of the construct, and post-printing maturation. At the initial stage, medical imaging data and computer-aided design tools are used to create a three-dimensional model of the target structure. This is followed by the printing stage, during which cells and biomaterials are spatially distributed according to a predefined architecture. The final stage involves cell proliferation, structural stabilization, and functional maturation of the engineered tissue. In cases where the construct is intended for therapeutic use, it may subsequently be implanted into the damaged area to restore biological function [2].

Technologically, 3D bioprinting relies on the coordinated interaction of hardware systems, specialized software, and biologically compatible materials. Modern bioprinters are equipped with automated deposition modules capable of precise positioning in three-dimensional space, while digital control systems regulate the geometry and internal organization of the printed construct. Bioinks are typically composed of aqueous, biocompatible materials that support cell viability and maintain structural integrity during and after printing [3].

Currently, the most widely used bioprinting approaches include extrusion-based, droplet-based, and laser-assisted techniques [4]. Extrusion systems enable the continuous deposition of viscous biomaterials, droplet-based technologies allow controlled micro-volume delivery, and laser-assisted methods provide high spatial precision through localized energy transfer. Continuous technological development has led to the emergence of hybrid material systems, improved construct stability, and expanded opportunities for personalized medical solutions. Bioprinting technologies are already applied in the fabrication of skin grafts, heart valve elements, cartilage structures, and bone tissue substitutes [5,6]. Experimental studies demonstrate that tissue-engineered constructs produced by additive technologies are capable of restoring not only anatomical integrity but also functional characteristics of damaged tissues [7].

One of the major challenges of modern transplantology is the persistent shortage of donor organs, which significantly limits the availability of life-saving therapies. In addition, conventional transplantation often requires lifelong immunosuppressive treatment, which increases the risk of complications. These limitations stimulate the development of alternative strategies within regenerative medicine aimed at creating biological substitutes capable of restoring tissue structure and function. Tissue engineering integrates cellular technologies and biomaterials to produce functional analogs of biological tissues under controlled laboratory conditions. Within this context, three-dimensional bioprinting represents a rapidly developing approach that enables the precise fabrication of complex tissue constructs adapted to individual patient characteristics.

Recent studies published between 2023 and 2025 demonstrate a clear shift toward advanced bioink formulations, multicellular construct design, and translational biofabrication strategies aimed at improving the clinical applicability of engineered tissues. The evolution of material science, cell engineering, and biofabrication technologies is gradually transforming experimental prototypes into more functionally integrated tissue models with increased therapeutic potential. Nevertheless, important challenges related to vascularization, long-term stability, standardization of production protocols, and regulatory approval pathways remain unresolved. The following sections examine the fundamental principles of 3D bioprinting, its technological approaches, biomedical applications, as well as current limitations and future directions of additive biofabrication research [8].

## 2. Principles and Stages of 3D Bioprinting

Three-dimensional bioprinting is based on the principle of layer-by-layer additive manufacturing, in which a three-dimensional structure is formed through the sequential deposition of material [9]. The bioprinting process is commonly divided into three main stages: pre-processing, processing (the actual printing stage), and post-processing [10].

Pre-processing. This stage includes the planning and design of the future tissue or organ construct. Medical imaging techniques, such as CT or MRI, are used to obtain a digital three-dimensional model of the target anatomical structure. The model is then processed in CAD systems and converted into a printer-readable format, such as an STL file. At this stage, the scaffold architecture is defined, and the appropriate biomaterials and cell types are selected. As a result, a digital blueprint is created that corresponds to the required dimensions and geometry of the defect.

Processing (Fabrication). During the printing stage, bioinks—composed of cells suspended in a hydrogel or another supporting matrix—are dispensed through the printhead in accordance with the digital model. A robotic system deposits the material layer by layer in predefined spatial coordinates along the X, Y, and Z axes, thereby forming a three-dimensional scaffold with a controlled distribution of cellular elements [11]. The maintenance of sterile conditions and preservation of cell viability, including control of temperature and pH, are critically important throughout this stage.

Post-processing. After fabrication, the printed biological construct undergoes maturation in a controlled environment or within a bioreactor, where cell proliferation, tissue remodeling, and differentiation continue until the required functional properties are achieved [12]. If the construct is intended for research purposes, it may be cultured in vitro for an extended period. If it is designed for therapeutic use, it may be implanted into the damaged area after a period of initial maturation. In both cases, the purpose of post-processing is to preserve cell viability, improve structural integrity, and promote the functional development of the engineered tissue prior to application [13].

**Figure 1:**
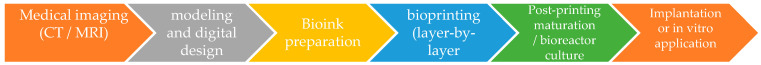
General workflow of 3D bioprinting, including medical imaging, CAD-based modeling, bioink preparation, layer-by-layer fabrication, maturation of the printed construct, and its subsequent experimental or clinical application.

The overall workflow of 3D bioprinting is presented schematically in Figure 1.

## 3. Key Components of a Bioprinter

Successful 3D bioprinting depends on the coordinated operation of three key components: hardware, software, and bioink. Together, these elements determine the precision of fabrication, the viability of printed cells, and the functional potential of the final construct.

Hardware (Equipment). The core of the system is the bioprinter itself—a robotic device capable of precise movement of the print head in three-dimensional space along the X, Y, and Z axes. The printer is equipped with a system for the controlled deposition of biomaterial. Depending on the type of bioprinter, this may involve syringe-based extruders, nozzles, or laser emitters that enable specific methods for depositing cells and hydrogels onto the substrate [14]. The hardware also includes temperature controllers to maintain optimal conditions for printing cell-laden materials, and in some cases integrated imaging or calibration systems. In intraoperative bioprinting, the hardware may additionally include mobile positioning mechanisms capable of operating directly over a wound site. All of these components must ensure sterility, positional accuracy, and reliable reproduction of the digital design.

Software. Specialized software controls the operation of the bioprinter by converting a digital three-dimensional model (e.g., an STL file) into a sequence of commands and spatial coordinates for the print head [15]. The software regulates the movement path of the print head, the feed rate of the material, the sequence of layer formation, and process parameters such as extrusion pressure or irradiation intensity during photopolymerization. It may also include modules for medical image segmentation and for the automated design of complex internal architectures, including pore geometry, vascular-like channels, and region-specific material distribution. Properly configured software enables the accurate positioning of different cell types and biomaterials within the construct, thereby facilitating the reproduction of the heterogeneous microstructure of native tissues [8].

Biological Material (Bioinks). Bioinks are suspensions of living cells within a biocompatible matrix suitable for printing. In most cases, the matrix is formed by hydrogels—aqueous polymer systems that mimic aspects of the extracellular matrix and support cell survival [16]. Bioinks must possess rheological properties that allow them to pass through the printhead while maintaining shape fidelity after extrusion or droplet deposition. Common components include natural polymers such as collagen, gelatin, alginate, fibrin, gellan gum, and hyaluronic acid, as well as hybrid formulations supplemented with synthetic polymers to improve mechanical stability [17]. A fundamental requirement is that bioinks must preserve cell viability and support cell attachment, proliferation, and differentiation after printing [18]. In addition to the matrix and cellular component, bioinks may contain biologically active molecules such as growth factors or signaling peptides that guide tissue-specific development. Because the requirements of each printing technique differ, bioinks must be tailored with respect to viscosity, gelation kinetics, and crosslinking behavior. Thus, 3D bioprinting operates at the intersection of engineering, materials science, and cell biology, requiring the integration of all three components into a unified and highly controlled process [19].

### 3.1. Advanced Bioink Design

In recent years, bioink development has moved beyond the simple combination of cells and supportive hydrogels toward more functionally specialized systems. Current studies increasingly focus on advanced bioink formulations capable of reproducing not only the structural but also the biochemical and mechanical features of native tissue microenvironments. Among the most promising approaches are decellularized extracellular matrix (dECM)-based bioinks, which retain tissue-specific biochemical cues and may improve cell adhesion, proliferation, and differentiation. Their use is particularly relevant for the fabrication of constructs intended to replicate the characteristics of highly specialized tissues.

Another important trend is the development of multicellular bioink systems containing two or more cell populations arranged to better reproduce the complexity of native tissues. Such formulations make it possible to model intercellular interactions more realistically and to create constructs with enhanced biological functionality. In parallel, increasing attention is being paid to stimuli-responsive bioinks, including materials sensitive to temperature, pH, or other environmental signals. These systems are of particular interest for advanced biofabrication strategies because they allow post-printing modulation of construct properties and are considered a promising basis for the further development of 4D bioprinting approaches.

The functional role of bioinks is therefore no longer limited to ensuring printability alone. Their composition directly influences cell behavior, matrix remodeling, and the regulation of the local microenvironment within the printed construct. For this reason, modern bioink design is increasingly regarded as a central determinant of tissue maturation, structural stability, and translational potential in bioprinting-based tissue engineering.

### 3.2. Biocompatible Materials Used in 3D Bioprinting

Biocompatible materials used in 3D bioprinting can be broadly divided into natural, synthetic, and hybrid systems. Natural materials, including collagen, gelatin, alginate, fibrin, and hyaluronic acid, are widely used because of their biological similarity to the extracellular matrix and their ability to support cell attachment and proliferation. However, many of them exhibit limited mechanical strength and often require additional crosslinking or structural reinforcement.

Synthetic polymers such as polyethylene glycol (PEG) and polycaprolactone (PCL) offer greater control over mechanical properties, degradation rate, and reproducibility. At the same time, their bioactivity is usually lower than that of natural materials, which may limit direct cell interactions unless they are combined with biologically active components. For this reason, hybrid systems have gained particular importance in recent years. By combining natural and synthetic components, hybrid bioinks make it possible to balance biological performance with structural stability.

The choice of material is ultimately determined by the target tissue, the printing strategy, and the desired post-printing performance of the construct. As a result, the selection of bioinks and supporting materials is no longer viewed as a purely technical task, but as a critical part of tissue-specific design in additive biofabrication. A summary of commonly used bioinks and biocompatible materials is presented in Table 1.

## 4. 3D Bioprinting Methods

Several technologies implement the principle of 3D bioprinting and are classified according to the method of material deposition and solidification. The main approaches include extrusion-based, inkjet (droplet-based), and laser-assisted bioprinting [20]. In addition to these established methods, several emerging strategies—such as acoustic, magnetic, microfluidic, and volumetric bioprinting—are currently under development, although most of them remain at an early experimental stage [21]. The three principal bioprinting methods are outlined below.

Extrusion-Based Bioprinting. Extrusion-based, or dispensing, bioprinting is currently the most widely used biofabrication technology. It creates three-dimensional structures through the continuous extrusion of bioink through a nozzle under mechanical pressure. The bioink typically consists of a viscous, cell-laden hydrogel loaded into a syringe or cartridge. Under pneumatic pressure, piston-driven force, or screw-assisted extrusion, the material is dispensed through a calibrated orifice and deposited as a continuous filament onto a substrate. Computer-controlled movement of the print head and/or platform along three axes allows these filaments to be arranged according to a predefined path, thereby generating complex three-dimensional structures layer by layer.

Extrusion-based technology is valued for its versatility and scalability, since it can process biomaterials with a wide range of viscosities (from approximately 30 mPa·s to 10^7^ mPa·s) and high cell densities. It is suitable for the fabrication of relatively large constructs and is technically straightforward compared with other methods, which contributes to its broad adoption. However, extrusion-based bioprinting also has limitations. Its spatial resolution usually does not exceed approximately 100 µm, and the passage of viscous materials through narrow nozzles may generate shear stress that damages cells and reduces viability [22]. Despite these constraints, extrusion remains the dominant method for producing many tissue-engineered constructs, including cartilage-like structures, skin substitutes, and cardiac tissue prototypes.

Inkjet (Droplet-Based) Bioprinting. Inkjet bioprinting, also referred to as drop-on-demand bioprinting, is based on the generation and controlled ejection of microdroplets of biofluid onto a substrate. Unlike continuous extrusion, the material is deposited in discrete volumes ranging from picoliters to nanoliters. Droplet size and velocity depend on the viscosity and surface tension of the bioink, the nozzle diameter, and the actuation mechanism. The three main droplet ejection mechanisms are thermal, piezoelectric, and electrostatic actuation.

In the thermal method, a micro-heater rapidly vaporizes a small volume of fluid, creating a vapor bubble that forces a defined quantity of bioink through the nozzle. Piezoelectric actuation relies on a piezoelectric crystal that deforms in response to an electric pulse and generates an acoustic wave that expels the droplet. In the electrostatic method, short high-voltage pulses accelerate droplets toward the substrate by electrostatic attraction.

Inkjet systems can deposit droplets with high positional accuracy and at high frequency. Their main advantages include printing speed, relative simplicity, and lower cost, since many systems are adapted from conventional inkjet platforms [23]. Another important advantage is the absence of direct mechanical contact between the nozzle and the substrate, which reduces the risk of contamination and minimizes cell damage. However, this method imposes strict requirements on bioink properties, as the material must remain sufficiently fluid and must not clog the micro-orifices [24].

In addition, the fabrication of dense or highly volumetric structures from individual droplets remains technically challenging, particularly when highly viscous materials are required. Nevertheless, inkjet bioprinting is highly effective for cell patterning and for the production of thin, multilayered tissues. For example, it can be used to deposit cells directly onto wound surfaces to generate skin grafts of irregular geometry. Experimental studies have shown that in situ inkjet bioprinting of skin can accelerate epithelialization and wound closure compared with conventional treatment approaches [24,25].

Laser-Assisted Bioprinting. Laser-assisted bioprinting includes two main strategies: photopolymerization-based printing, such as laser stereolithography (SLA), and laser-induced forward transfer (LIFT). Both are nozzle-free, non-contact deposition technologies and therefore avoid the problems of nozzle clogging and shear stress on cells [26].

Bioprinting via Photopolymerization (Laser Stereolithography, SLA). This method uses a liquid photosensitive polymer, typically a hydrogel containing a photoinitiator, which is selectively cured by a focused laser beam or projected light. By exposing defined regions of the polymer layer by layer to ultraviolet or visible light, the material is solidified into the desired three-dimensional geometry. To accelerate fabrication, a digital micromirror device (DMD) may be used to illuminate an entire layer simultaneously according to a digital mask, rather than scanning a single beam across the surface. The main advantages of SLA include very high precision and resolution, often reaching the micrometer range, as well as the absence of direct mechanical stress on embedded cells. However, the method requires suitable biocompatible photopolymers, and some photoinitiators or light sources may exert cytotoxic or mutagenic effects. In addition, the equipment is expensive, and the process remains relatively slow, which currently limits broader application [3].

Laser-Induced Forward Transfer (LIFT). LIFT is a nozzle-free transfer method in which short laser pulses are used to propel microdroplets of biomaterial from a donor surface to a receiving substrate. In a typical setup, a thin layer of bioink is spread onto a transparent donor ribbon, while the receiving substrate is positioned below it. A focused laser pulse generates localized evaporation at the donor interface, producing a microdroplet that is transferred onto the target surface. Repeated laser pulses allow the build-up of a patterned or three-dimensional structure. LIFT provides high spatial resolution and excellent positional control while avoiding nozzle-induced stress. It also accommodates a relatively broad range of material viscosities. High post-printing cell viability has been reported in studies involving delicate cell populations, including stem cells and neuronal cells [27]. At the same time, the method remains technically complex, expensive, and potentially vulnerable to thermal effects on sensitive biological components.

A comparative analysis of the principal 3D bioprinting techniques is presented in Table 2.

Although extrusion-based, inkjet, and laser-assisted bioprinting are the dominant methods, additional technologies continue to emerge. Acoustic bioprinting uses focused ultrasonic waves for the non-contact manipulation of droplets and enables the gentle assembly of cell-containing structures without nozzle interaction. Magnetic bioprinting employs magnetically responsive cells or hydrogels that can be spatially organized using an external magnetic field. Embedded bioprinting deposits soft bioinks into a supportive bath, which prevents structural collapse and allows the fabrication of complex overhanging geometries. Microfluidic bioprinting integrates the printhead with a microfluidic chip, enabling the real-time mixing of different materials and cell populations during fabrication. Volumetric bioprinting uses rotating light projections to generate entire three-dimensional structures in seconds by exposing the photopolymer from multiple directions simultaneously; small organ-like constructs have already been fabricated within 30–60 s using this approach [15].

Taken together, the major 3D bioprinting methods differ substantially in their balance between resolution, bioink compatibility, cell viability, scalability, and translational potential. Extrusion-based printing remains the most versatile and scalable approach for fabricating clinically relevant constructs, but its lower resolution and potential for shear-induced cell damage remain important limitations. Inkjet bioprinting offers high speed and precise droplet placement with relatively low mechanical stress, although it is restricted to low-viscosity bioinks and is less suitable for the fabrication of thick structures. Laser-assisted methods provide superior resolution and high post-printing cell viability, but their cost, technical complexity, and dependence on specialized materials limit widespread adoption. Consequently, no single method can currently satisfy all requirements of tissue engineering, and the selection of a bioprinting strategy must be guided by the specific biological, mechanical, and translational demands of the target tissue.

## 5. Applications of 3D Bioprinting in Medicine

The introduction of three-dimensional bioprinting technologies has had a significant impact across several areas of medicine [28]. 3D bioprinting has found applications in both fundamental research, including organ modeling and drug testing, and clinically oriented fields, ranging from the production of anatomical training models and pre-operative planning tools to the development of implants for reconstructive surgery and tissue substitutes for transplantation [29]. Key examples of bioprinting applications for different tissue and organ types are discussed below.

### 5.1. Anatomical Models and Surgical Planning

One of the earliest practical applications of 3D printing in medicine was the fabrication of patient-specific anatomical models based on medical imaging data using plastics or resins [30]. Bioprinting advances this concept by enabling the creation of models that reproduce not only the geometry but also selected biological properties of tissues. Such biomimetic models are used by surgeons for pre-operative planning of complex interventions, as they improve anatomical understanding and allow rehearsal of implant placement or technically demanding procedures [31].

A representative example is the use of 3D printing for planning transcatheter “valve-in-valve” replacement in the tricuspid position. In one study, a detailed 3D model of the heart and a degenerated tricuspid valve bioprosthesis was created from CT angiography data. The printed model allowed surgeons to rehearse the intervention, choose the appropriate valve size, determine stent positioning, and assess the risk of embolism or obstruction [32]. In a subsequent clinical series, all patients successfully underwent implantation via the femoral vein, with immediate elimination of regurgitation and improved hemodynamic performance [33]. These findings suggest that the integration of imaging data and 3D-printed patient-specific models can enhance procedural safety and support personalized treatment planning [34].

Beyond surgical planning, printed biomodels are also widely used in medical training. Students and early-career clinicians can study organ geometry, practice dissection, and rehearse suturing or procedural maneuvers without risk to patients. For example, heart models incorporating pathological features such as septal defects or aneurysms are used in cardiac surgery education to simulate realistic operative conditions. Bioprinting enables such models to be fabricated from hydrogels with tissue-like consistency and may incorporate vessel- or valve-like elements, thereby improving training realism [35].

### 5.2. Skin and Soft Tissues

Skin regeneration remains one of the most urgent challenges in reconstructive medicine, particularly in combustiology and the treatment of chronic wounds [36]. 3D bioprinting of skin has already produced promising results. Several experimental skin bioprinters have been developed for the direct deposition of skin equivalents onto wound surfaces. For example, a portable bioprinter integrated with a wound scanner was designed to map the contours and depth of a defect and then deposit a suspension of dermal fibroblasts and epidermal keratinocytes in a hydrogel directly onto the wound bed [37,38]. In a preclinical porcine model, wounds treated in this manner showed significantly faster epithelial coverage, with near-complete closure within 2–3 weeks, whereas standard treatment required more than 5 weeks for comparable epithelialization [39]. The printed skin grafts formed organized epidermal and dermal layers with minimal scar formation.

Another direction is the production of in vitro skin flaps. Lee et al. used extrusion-based bioprinting to fabricate a hydrogel sheet containing multilayered keratinocytes in the upper region and fibroblasts in the lower region, thereby reproducing the organization of the epidermis and dermis [40]. The printed cells remained viable and proliferative. Subsequent studies demonstrated that similar skin constructs could be grafted onto wound surfaces in animals. In one porcine burn model, bioprinted full-thickness skin patches showed accelerated graft integration, earlier vascularization, and significantly faster wound closure than conventional autografts [41]. These results indicate that bioprinting can generate skin substitutes of predetermined shape and thickness while reducing healing time and limiting complications. Current work focuses on refining skin bioinks by incorporating pro-angiogenic factors and improving the mechanical stability of printed skin constructs [42].

### 5.3. Cartilage Constructs

Cartilage possesses very limited regenerative capacity, and defects affecting articular cartilage or auricular structures often require surgical replacement. Conventional prosthetic options remain suboptimal in terms of biological integration and long-term performance. 3D bioprinting has enabled the production of cartilage constructs with complex geometry and tissue-like flexibility.

A notable achievement in this field is the printing of architecturally complex structures such as the auricle and meniscus. Zenobi-Wong and colleagues developed a biodegradable bioink composed of gellan gum and alginate supplemented with cartilage-derived extracellular matrix particles [43]. These materials demonstrated suitable viscosity and mechanical properties for printing. Using this formulation, the authors reproduced human ear cartilage and meniscal structures with complex curvilinear geometry [44]. After bioreactor-based maturation, the constructs developed a dense cartilaginous matrix. Biomechanical testing demonstrated elasticity and strength close to native auricular cartilage, while implantation in rabbits showed good biocompatibility, minimal inflammation, and maintenance of the original shape. These findings suggest strong potential for individualized cartilage replacement.

Another important direction involves the fabrication of osteochondral constructs for articular cartilage repair. In such systems, a cartilage-forming bioink is deposited in the upper layer, while a mineral-containing or hydroxyapatite-based formulation forms the lower osseous component. Preliminary animal studies have shown that these bilayer implants can restore the articular surface while integrating with the underlying bone [45]. Their major advantage lies in their precise geometric adaptation to irregular defects based on imaging-guided design.

### 5.4. Bone Tissue

The regeneration of large bone defects remains a major clinical challenge because autografts and donor bone are associated with several limitations, including donor-site morbidity, limited availability, and incomplete integration [46]. 3D bioprinting offers a strategy for creating bone scaffolds precisely matched to the geometry of the defect and seeded with osteogenic cells. These constructs may be fabricated from synthetic materials, ceramic composites, or cell-laden hydrogel systems [47].

A representative example is the fabrication of bone grafts using a composite of polycaprolactone (PCL) and hydroxyapatite (HA). In one study, digital 3D models of missing bone fragments were created from CT data, after which porous PCL-HA scaffolds were printed by extrusion to match the defect geometry [48]. When implanted into rabbit femoral defects and lumbar vertebral defects, these constructs showed excellent geometric fit and promoted the guided growth of new bone tissue. Over time, bone formed within the porous scaffold, and the regenerated tissue approached normal mechanical performance [49]. Additional studies have shown that stem cell seeding and the incorporation of osteogenic factors may further enhance the regenerative potential of such constructs [50]. These findings support the use of bioprinting for personalized bone repair.

### 5.5. Cardiac Tissue and Vasculature

The cardiovascular system remains one of the most technically demanding targets for 3D bioprinting because of its structural complexity and the need for coordinated mechanical and electrophysiological function. Nevertheless, notable progress has been made in the printing of heart valves, myocardial constructs, and vascular grafts.

Bioprinted heart valves are of particular interest because conventional prostheses have significant limitations: mechanical valves require lifelong anticoagulation, while biological valves undergo structural degeneration over time. Bioprinting offers the possibility of producing individualized valve constructs adapted to patient anatomy and potentially capable of remodeling after implantation [51]. Experimental studies have shown that printed valve structures may achieve favorable hemodynamic performance and low thrombogenicity when fabricated from appropriate polymeric materials [52,53]. However, the long-term durability and remodeling behavior of such constructs remain insufficiently understood.

Significant advances have also been reported in the bioprinting of contractile myocardium. In 2023, researchers developed a miniature left ventricular model using a fiber-infused gel (FIG) bioink in which fine gelatin fibers guided the alignment of cardiomyocytes during printing [33]. The resulting construct displayed coordinated contraction and fluid ejection under electrical stimulation. Although such models remain limited in contractile force and long-term function, they represent an important step toward engineered cardiac patches and in vitro models for cardiotoxicity testing [54].

The fabrication of vascular structures is another major area of application. Large-caliber vessels have been printed using coaxial extrusion systems that generate tubular constructs with layered cell organization. Such grafts have shown the ability to withstand physiological pressure in vitro and remain patent after implantation in animal models [55]. For smaller vascular networks, methods such as stereolithography and sacrificial templating have been explored to create channel systems that support perfusion and vessel ingrowth. The problem of vascularization remains a major bottleneck for thick tissue constructs, but current approaches increasingly focus on incorporating vascular channels directly into the printed architecture [56].

### 5.6. Skeletal Muscles

The restoration of skeletal muscle after trauma or oncological resection requires the reconstruction not only of tissue volume but also of aligned and functional muscle architecture. 3D bioprinting enables the fabrication of scaffolds that direct the spatial orientation and maturation of muscle cells [57].

Costantini et al. demonstrated a microfluidic extrusion strategy for printing aligned muscle constructs using a PEG-fibrinogen-based bioink containing myoblasts and fibroblasts [21]. The resulting structure consisted of parallel hydrogel filaments in which cells aligned linearly. After several days of in vitro culture, the myoblasts fused into multinucleated myotubes and formed muscle-like fibers with sarcomeric organization. When implanted subcutaneously into immunodeficient mice, the constructs retained muscle-specific markers and showed integration with the host tissue. These findings indicate that bioprinting can support the formation of organized skeletal muscle and may eventually contribute to the development of functional muscle grafts.

### 5.7. Nervous Tissue

Three-dimensional bioprinting is also being explored for peripheral nerve repair. In cases of major nerve loss, autografts remain the clinical standard, but their availability is limited and functional recovery is often incomplete. Bioprinting enables the fabrication of nerve conduits that guide axonal regeneration while matching the size and geometry of the defect.

In one recent study, a living nerve conduit was fabricated from umbilical cord-derived stromal cells arranged into a cylindrical construct and incorporated into a hydrogel scaffold corresponding to the shape of the missing nerve segment [58]. Implantation in a rat sciatic nerve defect model resulted in axonal regrowth and partial restoration of limb function within several weeks [59]. Although such approaches remain preclinical, they demonstrate the potential of personalized nerve implants designed to support guided regeneration without the need for conventional graft harvesting.

### 5.8. Reproductive Organs

An emerging and particularly innovative direction is the application of bioprinting to reproductive tissue engineering. One of the most notable examples is the fabrication of ovarian constructs intended to restore endocrine and reproductive function in patients with ovarian insufficiency.

Researchers have proposed the creation of a porous ovarian scaffold from a biodegradable hydrogel into which immature follicles are seeded. In a mouse model, implantation of a printed ovarian construct enabled previously sterilized animals to regain fertility and produce healthy offspring [33]. In this system, a gelatin-based bioink was used to generate a porous scaffold with approximately 0.5 mm pores, allowing follicle placement and vascular access [60]. After implantation, the follicles progressed through maturation, endocrine activity was restored, and pregnancy occurred in a subset of the treated animals [61,62]. These results demonstrate that bioprinting may eventually provide a means of restoring both fertility and hormonal function. At present, however, this field remains highly experimental and depends on further progress in vascularization and long-term construct integration.

Taken together, these examples show that 3D bioprinting is no longer limited to proof-of-concept fabrication, but is increasingly being used to address tissue-specific biomedical tasks, including structural replacement, functional modeling, regenerative support, and personalized therapeutic design. The translational relevance of these studies varies across tissue types: applications involving skin, cartilage, bone, and vascular constructs are currently closer to practical implementation, whereas cardiac, neural, and reproductive applications remain more dependent on further advances in vascularization, long-term functionality, and regulatory validation. At the same time, recent studies published in 2023–2025 indicate a clear shift toward more application-oriented biofabrication strategies, including multicellular constructs, tissue-specific bioinks, and patient-adapted design approaches. Representative examples of major biomedical applications of 3D bioprinting are summarized in Table 3.

## 6. Challenges and Future Prospects

Despite substantial progress, 3D bioprinting remains far from routine clinical implementation. Although a wide range of tissue-engineered constructs have already been developed in experimental and preclinical settings, several major barriers still limit broader translational use.

### 6.1. Vascularization

One of the central challenges in 3D bioprinting is the vascularization of large tissue constructs. Thick living tissues, generally exceeding 1–2 mm, require a functional blood supply; otherwise, cells located in the inner regions rapidly undergo hypoxia and nutrient deprivation. Effective vascularization requires not only the formation of internal channels, but also the successful integration of these channels with the host circulatory system after implantation. Current strategies include the printing of perfusable microchannels, the incorporation of endothelial cells, and the use of perfusion bioreactors to support tissue maturation before implantation [63]. Although these approaches have improved the viability of bioprinted tissues, the fabrication of stable capillary-scale networks remains a major unresolved issue. The development of methods capable of producing vascular systems that are both structurally complex and functionally connected to host circulation is likely to remain a priority in the coming years.

### 6.2. Functional Integration and Innervation

Another major limitation concerns the functional integration of printed tissues. Current bioprinted constructs are still relatively simple when compared with native organs. While tissues such as skin, cartilage, or small muscle fragments can be reproduced with increasing accuracy, more complex organs require a far more sophisticated internal organization involving multiple cell types, vascular structures, and neural elements. In particular, proper innervation remains a serious challenge. The integration of nerve endings is essential for the normal function of muscle tissue, sensory structures, and several other organ systems. Experimental approaches include the incorporation of neuronal cells or neurotrophic factors into bioinks, as well as the use of guidance structures to direct neural growth [64]. However, the formation of stable and functionally relevant neural networks within thick tissue constructs remains difficult. Future progress in this area will likely depend on hybrid strategies combining bioprinting with nerve grafting, bioactive signaling systems, or external stimulation.

### 6.3. Mechanical Properties and Scalability

The mechanical performance of printed constructs also remains a limiting factor. Many hydrogel-based bioinks are biologically favorable but mechanically weak, making them unsuitable for load-bearing applications without additional reinforcement. As a result, printed constructs may lack the stiffness, elasticity, or long-term stability required for implantation in vivo. This problem is especially relevant for tissues such as cartilage, bone, and vascular grafts, where mechanical integrity is essential [65]. At the same time, scalability presents a separate but equally important challenge. Current systems perform well for small- or medium-sized samples, but the fabrication of human-scale organs remains technically difficult. Printing large constructs may require many hours or even days, which creates additional problems related to cell survival, structural stability, and reproducibility [66]. Current solutions include the development of stronger composite materials, faster printing systems, multi-nozzle platforms, and volumetric bioprinting approaches capable of forming entire structures within a much shorter time frame.

### 6.4. Standardization and Quality Control

For 3D bioprinting to become clinically viable, reproducibility and quality control must be significantly improved. At present, different research groups use their own materials, printing parameters, and maturation protocols, which makes direct comparison difficult and slows the development of unified clinical standards. Standardized criteria are needed for bioink formulation, post-printing cell viability, structural fidelity, and mechanical testing of printed constructs. Without such standards, regulatory approval remains highly challenging [67]. In response to this problem, some research groups are developing automated monitoring systems that assess the printing process in real time through optical imaging, pressure control, and other sensor-based tools. These approaches may improve consistency and reduce technical defects, but they have not yet become universal. The establishment of robust and widely accepted quality-control frameworks will be essential for clinical translation.

### 6.5. Ethical and Regulatory Issues

Ethical and regulatory concerns represent another major obstacle to the wider use of bioprinted tissues and organs. The fabrication of human-derived biological structures raises questions that extend beyond technical feasibility. These include the status of constructs produced from donor-derived or engineered cellular material, the acceptability of highly complex organoid models, and the classification of bioprinted products within existing regulatory systems [68]. It is still unclear in many cases whether a printed construct should be treated as a medical device, a biological product, or a hybrid therapeutic platform. Questions of responsibility are equally important: if a personalized printed implant fails after clinical use, legal accountability may involve multiple participants, including physicians, engineers, and manufacturers. Furthermore, immunological safety remains a significant concern. Even when autologous cells are used, residual culture components, biomaterial degradation products, or incomplete maturation may still provoke unwanted biological responses [16,69]. For these reasons, future progress will require not only technical refinement, but also the development of clear legal definitions, ethical boundaries, and regulatory pathways.

### 6.6. Future Perspectives: 4D Bioprinting and Intelligent Design

Among the most promising future directions is the development of 4D bioprinting, in which printed constructs are capable of changing their shape or functional properties over time in response to external or internal stimuli such as temperature, moisture, pH, or electrical signals [70]. In the biomedical context, this may allow the creation of adaptive implants capable of responding to physiological conditions after implantation. Shape-memory hydrogels, stimuli-responsive scaffolds, and dynamically reconfigurable materials are already being explored for such purposes. In parallel, the integration of artificial intelligence into bioprinting workflows may substantially improve design accuracy and reproducibility. AI-assisted modeling has the potential to optimize scaffold geometry, predict material behavior, and improve process control, thereby reducing trial-and-error approaches in construct fabrication. Together, 4D bioprinting and intelligent design strategies may define the next stage in the evolution of additive biofabrication.

Overall, 3D bioprinting has progressed within a relatively short period from experimental demonstration to the development of tissue-engineered constructs with clear translational promise. Skin, cartilage, bone, and muscle substitutes have already shown encouraging results in preclinical models, while more complex applications involving vascularized or organ-level constructs are moving gradually closer to practical use. Even so, the field still depends on advances in vascular integration, material science, manufacturing standardization, and regulatory approval. Continued collaboration between engineers, biologists, clinicians, and regulatory specialists will determine how rapidly 3D bioprinting moves from the laboratory to routine medical practice.

## 7. Conclusions

The analysis presented in this review shows that additive biofabrication has become one of the most important directions in contemporary tissue engineering and regenerative medicine. Three-dimensional bioprinting makes it possible to fabricate biologically active and structurally complex constructs with a level of geometric precision that is difficult to achieve using conventional manufacturing approaches. The development of this field has been driven not only by advances in printing hardware and digital modeling, but also by significant progress in bioink design, biomaterials engineering, and cell-based fabrication strategies.

The current range of biomedical applications demonstrates the broad potential of 3D bioprinting. Promising results have already been reported in the fabrication of skin, cartilage, bone, vascular, muscular, neural, and reproductive tissue constructs, as well as in the development of patient-specific anatomical models for surgical planning and medical training. At the same time, the translational maturity of these applications remains uneven. While some directions, particularly skin, cartilage, and bone reconstruction, are moving closer to practical implementation, more complex organ-level systems still depend on further advances in vascularization, long-term functionality, and biological integration.

Particular importance should be assigned to the development of advanced bioinks and biocompatible material systems. The transition from simple hydrogel-based formulations to tissue-specific, multicellular, and stimuli-responsive bioinks has significantly expanded the biological functionality of printed constructs. This shift indicates that the success of bioprinting increasingly depends not only on printing accuracy, but also on the ability to reproduce the biochemical and mechanical features of the native cellular microenvironment.

Despite these achievements, major challenges remain unresolved. The most significant limitations involve the vascularization of large tissue constructs, the recreation of functionally integrated multicellular systems, the mechanical stability and scalability of printed products, and the lack of standardized manufacturing and quality-control protocols. In addition, ethical and regulatory issues continue to influence the pace of clinical translation.

Future progress in this field is likely to be closely associated with the development of 4D bioprinting, intelligent design strategies, and more predictive computational approaches for construct optimization. The integration of additive manufacturing, materials science, cell engineering, and digital modeling is steadily transforming 3D bioprinting from an experimental platform into a clinically relevant biofabrication technology. Although the routine production of fully functional organs remains a long-term goal, the current trajectory of research clearly indicates that additive technologies will continue to redefine the possibilities of regenerative medicine.

## Figures and Tables

**Table 1:** Common bioinks and biocompatible materials used in 3D bioprinting and their key properties.

Material/Bioink	Type	Key Properties	Advantages	Limitations	Typical Applications
Collagen	natural polymer	high biocompatibility, ECM-like structure	supports cell adhesion	low mechanical strength	skin, cartilage
Gelatin	natural polymer	biodegradable, thermosensitive	good printability	weak structural stability	soft tissues
Alginate	natural polymer	fast gelation, good viscosity	easy crosslinking	low cell adhesion	cartilage, bone
Fibrin	natural polymer	promotes cell migration	good biological activity	low mechanical strength	vascular tissue
Hyaluronic acid	natural polymer	ECM component	supports cell proliferation	requires modification	skin, cartilage
GelMA	modified natural polymer	tunable mechanical properties	good cell viability	requires UV crosslinking	various tissues
PEG	synthetic polymer	controllable stiffness	reproducibility	low bioactivity	structural scaffolds
PCL	synthetic polymer	high mechanical strength	long degradation time	low cell affinity	bone tissue
dECM-based bioinks	natural-derived	tissue-specific signals	promotes differentiation	complex preparation	organ-specific tissues
Multicomponent bioinks	hybrid systems	tunable properties	improved functionality	complex formulation	complex tissues

**Table 2:** Comparative analysis of principal 3D bioprinting methods used in tissue engineering.

Method	Resolution	Cell Viability	Viscosity Range	Advantages	Limitations	Typical Applications
Extrusion-based bioprinting	moderate (~100 µm)	moderate to high, but may decrease under shear stress	broad (from low to very high)	scalability, versatility, ability to print high-viscosity materials and high cell densities	lower resolution, nozzle-induced shear stress	cartilage, bone, skin, cardiac tissue
Inkjet bioprinting	high	high under optimized conditions	low	rapid printing, low cost, precise droplet deposition, reduced mechanical stress	limited to low-viscosity bioinks, difficulty in fabricating thick structures, nozzle clogging	skin, cell patterning, thin multilayered tissues
Laser-assisted bioprinting	very high	high	moderate to broad	high precision, nozzle-free deposition, minimal shear stress	high cost, technical complexity, limited accessibility, possible thermal effects	vascular structures, neural constructs, microscale tissue architectures

**Table 3:** Representative biomedical applications of 3D bioprinting and their translational relevance.

Tissue Type	Bioprinting Method	Bioink/Material	Experimental Model	Key Outcome	Translational Relevance
Anatomical models	extrusion/model-based printing	polymers, hydrogels	patient-specific cardiovascular models	improved surgical planning, risk reduction	high relevance for personalized surgery
Skin	inkjet/extrusion	fibroblasts, keratinocytes, hydrogels	porcine wound models	accelerated epithelialization, reduced scarring	close to clinical application
Cartilage	extrusion	alginate, gellan gum, ECM-based bioinks	rabbit implantation	shape fidelity, mechanical similarity to native cartilage	promising for auricular reconstruction
Bone	extrusion	PCL, hydroxyapatite composites	rabbit bone defects	guided bone regeneration, good integration	high relevance for patient-specific grafts
Cardiac tissue	extrusion/laser-assisted	hydrogel cardiac bioinks, FIG ink	ventricular tissue models	synchronized contraction, perfusable constructs	promising but limited by long-term stability
Skeletal muscle	microfluidic extrusion	PEG-fibrinogen bioinks	mouse implantation	aligned myofibers, structural organization	potential for functional muscle repair
Nervous tissue	scaffold-based bioprinting	hydrogel nerve conduits, UC-MSC constructs	rat sciatic nerve injury model	axonal regeneration, partial functional recovery	promising for peripheral nerve repair
Reproductive tissue	scaffold-based bioprinting	gelatin-based porous hydrogels	mouse ovarian failure model	restoration of fertility and endocrine activity	innovative but experimental direction

## Data Availability

No new data were created or analyzed in this study.

## Abbreviations

3D	Three-Dimensional
CAD	Computer-Aided Design
MRI	Magnetic Resonance Imaging
CT	Computed Tomography
SLA	Stereolithography
LIFT	Laser-Induced Forward Transfer
PCL	Polycaprolactone
HA	Hydroxyapatite
UC-MSCs	Umbilical Cord Mesenchymal Stem Cells
